# Early life microbial colonization of the gut and intestinal development differ between genetically divergent broiler lines

**DOI:** 10.1186/s12864-015-1646-6

**Published:** 2015-05-28

**Authors:** Dirkjan Schokker, Gosse Veninga, Stephanie A. Vastenhouw, Alex Bossers, Freddy M. de Bree, Lucia M. T. E. Kaal-Lansbergen, Johanna M. J. Rebel, Mari A. Smits

**Affiliations:** Wageningen Livestock Research, Wageningen, The Netherlands; Cobb Europe BV, Boxmeer, The Netherlands; Central Veterinary Institute, Lelystad, The Netherlands

**Keywords:** Gut, Chicken, Immune, Microbiota, Gene expression

## Abstract

**Background:**

Host genetic makeup plays a role in early gut microbial colonization and immune programming. Interactions between gut microbiota and host cells of the mucosal layer are of paramount importance for a proper development of host defence mechanisms. For different livestock species, it has already been shown that particular genotypes have increased susceptibilities towards disease causing pathogens.

The objective of this study was to investigate the impact of genotypic variation on both early microbial colonization of the gut and functional development of intestinal tissue. From two genetically diverse chicken lines intestinal content samples were taken for microbiota analyses and intestinal tissue samples were extracted for gene expression analyses, both at three subsequent time-points (days 0, 4, and 16).

**Results:**

The microbiota composition was significantly different between lines on each time point. In contrast, no significant differences were observed regarding changes in the microbiota diversity between the two lines throughout this study. We also observed trends in the microbiota data at genus level when comparing lines X and Y. We observed that approximately 2000 genes showed different temporal gene expression patterns when comparing line X to line Y. Immunological related differences seem to be only present at day 0, because at day 4 and 16 similar gene expression is observed for these two lines. However, for genes involved in cell cycle related processes the data show higher expression over the whole course of time in line Y in comparison to line X.

**Conclusions:**

These data suggest the genetic background influences colonization of gut microbiota after hatch in combination with the functional development of intestinal mucosal tissue, including the programming of the immune system. The results indicate that genetically different chicken lines have different coping mechanisms in early life to cope with the outside world.

**Electronic supplementary material:**

The online version of this article (doi:10.1186/s12864-015-1646-6) contains supplementary material, which is available to authorized users.

## Background

During the last decades, growth performance traits were the main driver for the genetic selection programs applied for broilers. This already underscores the importance of genetic factors contributing to this important economic trait for poultry production. More recently the knowledge that intestinal microbiota also play a key role in growth-related traits such as feed digestibility, feed uptake, protein fermentation, but also in health related traits such as immune competence and immune tolerance. For human and mice data have been described that suggest a role for early-life colonizing microbiota in host immune programming [1–5], yet for chicken such data are lacking. Furthermore the impact of genotypic variation on early life microbial colonization in relation to the functional development of the gut is largely unknown. In fact, the gut microbiota is regarded a metabolic powerhouse that provides the functionally limited host with an extensive array of enzymes and substrates required for growth. It has been shown that gut microbiota can influence energy retention and can predispose to obesity [6, 7]. Additionally, they play a role in the renewal of gut epithelial cells and its barrier function [8, 9], the breakdown of toxins [10], the exclusion of pathogens [11], and the programming and development of the immune system [12]. The aim of this research was to investigate the impact of genetic background of these broiler lines on the microbial colonization and the development of the small intestine in early life.

In chickens, microbiota colonization occurs immediately after hatch, both microbiota from the egg shell and environment form the first inoculum of the chickens [13]. The characteristics of this first inoculum is of utmost importance because it impacts the further colonization of microbiota and simultaneously the functional development of the intestinal tissue in terms of barrier function and in terms of immune programming [13, 14]. It has been shown that around day 14 of age a presumably more stable microbiota is not yet established [15], however, immunological development in the small intestine has already occurred [16–18]. Around day 14, intestinal segments have different functional properties and harbour distinctive microbiota compositions [19].

In chickens, not much is known about the effect of host genetic background on microbial colonisation and microbiota composition. It has, however, been shown that high variation occurs in microbial composition between individual chickens and flocks of genetically closely related animals [20]. For mice it has been demonstrated that the host genetic background has impact on the microbiota composition in the gut [21]. However, it still difficult to disentangle the relationship between host genetic factors and the microbiota composition directly [22]. Nevertheless, first attempts have been made in mice to search for quantitative trait loci (QTL) associated to the presence and/or abundance of specific bacterial species or taxa and evidence is provided that host genetic control occurs in shaping the microbiota composition [23].

In poultry, not much is known about the effect of the genetic background on intestinal immune development. It is known that at hatch the innate and adaptive immune systems are immature and functional maturation occurs mainly the first 2 weeks of life in broilers [16, 18]. The innate immunity is the first line of defence, which includes the barrier function (epithelial layers), complement and coagulation cascade, phagocytes (e.g. macrophages), natural killer cells, and dendritic cells. Besides the role of the epithelial layer being the first line of defence against invading pathogens, these cells also play an important role in maintaining intestinal homeostasis by integration of microbial signals (reviewed in [24]). The adaptive immune system is also described as the acquired immune system, in which memory cells are generated after the initial challenge with an antigen. These memory cells facilitate a more efficient and faster response upon subsequent challenges with the same antigen.

It has been shown that restricting microbial exposure of chicken during early life has impact on mucin production [25]. Mucins are key components of epithelial layers, serving functions from lubrication to cell signalling to forming barriers to chemicals and pathogens. These observations already imply that bacterial colonization and critical aspects of gut (immune) homeostasis and/or barrier functions are intertwined with each other. Furthermore, studies on the effects of different pig rearing environments showed a negative effect of more ‘hygienic’ environments during early life on immune development [26]. Similarly, early life use of antibiotics in piglets has been shown to alter microbiota colonization as well as immune development [27]. It is proposed that a proper immune development requires colonization with “natural” microbiota present in the future environment [28].

The objective of this study was to investigate the impact of genetic background on both early microbial colonization of the gut in combination with functional development of intestinal tissue. To accomplish this, community-scale analysis of gut lumen microbiota and genome-wide transcriptome profiling of intestinal tissue was used. In this study we compared two genetically divergent chicken lines that differ from each other in health (immune) related phenotypes, in particular in bacterial infections and the related pathology and severity of such induced diseases by differing in immune response against *Salmonella enteritidis*, vancomycin-resistant Enterococcus, and *Campylobacter jejuni* [29–35]. The justification for using these selected lines was that they had known differences in susceptibility towards bacterial infections and thus may differ as well in the basic level of immune competence. To identify similar and dissimilar functional processes in time between the two chicken lines, the temporal gene expression profiles were analysed in more detail.

## Results

### Performance data

**Fig. 1:**
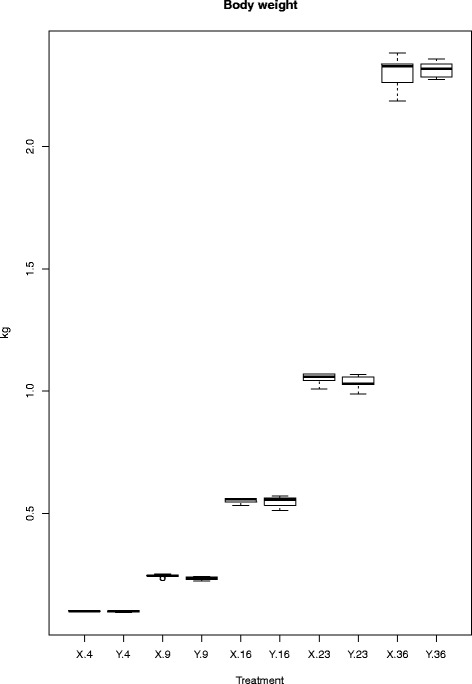
Boxplot of body weight in time for each chicken line. On the x-axis the lines X and Y and the days are depicted and the y-axis shows the body weight in kilograms. The letters X and Y stand for the chicken line and the numbers for the age in days

Body weight (Fig. 1) and Feed Conversion Ratio (FCR; Table 1) were measured in time, in order to generate a representative view of the whole life of a broiler. No statistical significant differences were observed per time-period between lines X and Y for both body weight and FCR. Body weight increased from approximately 100 grams on day 4 to approximately 2.3 kg on day 36. The FCR was slightly higher in line X compared to line Y at each time-point.

**Table 1:** Difference in feed conversion ratios in time between chicken line X and Y

Time-period	Line Y - X	Ratio in %
0-9	−0.004	99.46
9-16	−0.037	96.93
16-23	−0.001	99.91
23-36	−0.001	99.91

### Microbiota analyses

**Fig. 2:**
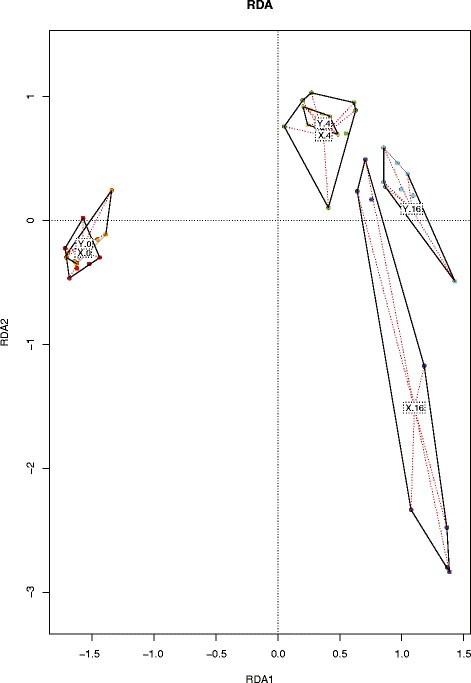
Redundancy analysis of jejunal microbiota composition (family level) on day 0, 4, and 16. Each symbol represents a pool of 10 chickens and represents their average microbiota composition. The data are represented as follows, for line X day 0 (*red*), day 4 (*green*), day 16 (*blue*) and for line Y day 0 (*orange*), day 4 (*light green*), day 16 (*cyan*)

**Fig. 3:**
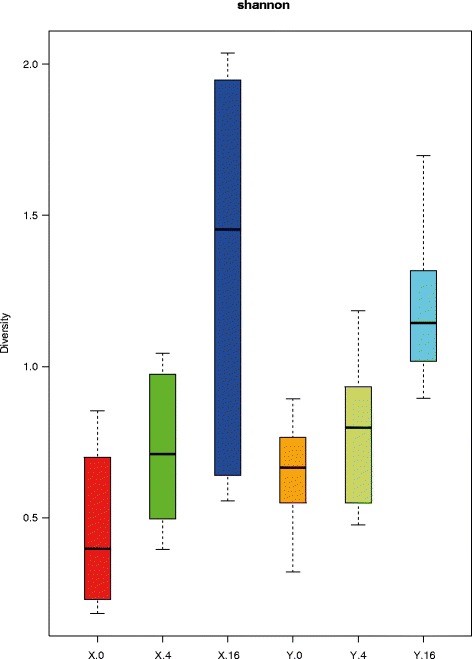
Microbiota diversity analysis of jejunal microbiota composition on day 0, 4, and 16. Box plot of the microbiota diversity measured by the Shannon index. The data are represented as follows, for line X day 0 (*red*), day 4 (*green*), day 16 (*blue*) and for line Y day 0 (*orange*), day 4 (*light green*), day 16 (*cyan*)

To investigate the differences between the two broiler lines regarding their microbiota, both redundancy (Fig. 2) and diversity analyses (Fig. 3) were performed. The redundancy analysis showed that the primary discriminant on the microbiota composition between the two genetically different broiler lines was the factor time, and secondary was the influence of genotype. An ANOVA permutation test for RDA under a reduced model, where terms were added sequentially (first to last) showed that Age, Line, and Age:Line were all significant, p-values 0.01, 0.03, and 0.01, respectively.

In the diversity analysis, which was calculated by the Shannon-index, we observed an increase in time of the microbiota diversity for each chicken line. However, no statistically significant differences were observed between the lines, in other words similar levels of diversity were observed between the two lines (X and Y).

To investigate to what extent the relative abundance of specific bacterial species were different between the chicken lines, averages of microbial families and/or species were calculated for each time-point separately (Table 2). At day 0 lines X and Y have different abundance levels of the 2 to 3 most dominant bacterial genera, *Enterococci, Escherichia* and *Lactobacilli*. Day 4 after hatch shows that the abundance of bacterial genera are more closely together, and the highest abundance is of *Lactobacilli*, 88 % average relative contribution (ARC) in line X and 84.5 % ARC in line Y. Also at day 16 similar ARC abundance at the bacterial genera level were observed between lines X and Y, however line Y had two specific genera, *Enterococci* and *Escherichia*, which were markedly higher compared to line X. Yet line X displayed overall higher ARC in various bacterial genera.

**Table 2:** Most abundant microbiota^a^ between lines X and Y

P^b^	Class	Family	Genus	X.0	Y.0	X.4	Y.4	X.16	Y.16
F	Bacilli	Enterococcaceae	Enterococcus	**83.5**	**71**	0.16	1.33	0.09	**0.72**
			Other	1.12	1.28	**7.74**	**8.6**	**3.2**	**6.12**
		Lactobacillaceae	Lactobacillus	0.12	0.02	**88**	**84.5**	**68.8**	**72.9**
		Streptococcaceae	Streptococcus	0	0	1.6	2.45	**14**	**15.1**
		Bacillaceae	Other	0	0	0	0	**1.48**	0.01
	Clostridia	Other	Other	0	0	0.14	0.09	**0.926**	0.04
		Lachnospiraceae	Other	0	0	0.12	0.02	**1.44**	0.11
		Ruminococcaceae	Faecalibacterium	0	0	0	0	**0.741**	0.01
	Erysipelotrichi	Erysipelotrichaceae	Coprobacillus	0	0	0.02	0	**1.5**	0.01
Pr	Gammaproteobacteria	Enterobacteriaceae	Escherichia	**12.6**	**26.8**	1.47	**2.69**	**1.98**	**0.84**
T	Mollicutes			0	0	0	0	**1.26**	0.03

^1^above 0.1 % average relative contribution in at least one condition

^2^P, Phylum; F, Firmicutes; Pr, Proteobacteria; T, Tenericutes

^3^In bold make up >95 % of the average relative contribution in a certain condition

### Transcriptome analyses

**Fig. 4:**
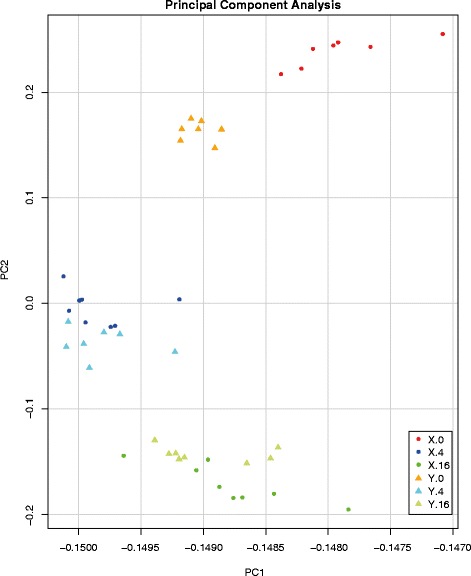
Principal Components Analysis of jejunal transcriptomics data of chicken lines X and Y. Each symbol represents whole-genome gene expression data (approximately 44 k probes) of a pool of 10 chickens. The data are represented as follows, for line X (*circles*) day 0 (*red*), day 4 (*blue*), day 16 (*green*) and for line Y (*triangles*) day 0 (*orange*), day 4 (*cyan*), day 16 (*light green*)

First an explorative principal component analysis (PCA) was performed, in which the different time-points and broiler lines could be separated (Fig. 4). In this analysis only the first two components were taken into consideration, because the variance explained was already 98.05 %. This PCA shows a convergence in overall gene expression in time between the two lines. At day 0, lines X and Y are clearly separated on both principal component 1 (PC1) and PC2, whereas on day 4 and 16 separation of the two lines was mainly on PC2.

To get more insight into differentially expressed genes between the two lines on each time-point, statistical testing by LIMMA was performed in which up- and down-regulation was also taken into account (Table 3; upper part). Subsequent to the LIMMA analysis, functional annotation clustering (DAVID) was performed to highlight particular processes that differ between lines X and Y (Table 3; lower part). All enrichment scores (ES) above 1.3 of a particular functional annotation cluster were taken into account for day 0, 4, and 16 separately. Each ‘enriched’ cluster was summarized manually into two-three representative keywords describing the generic term (Table 4).

**Table 3:** Differential expression between groups and corresponding functional annotation clustering

LIMMA	Y.0-X.0		Y.4-X.4		Y.16-X.16	
	Down	Up	Down	Up	Down	Up
Number of probes^a^	283	741	70	449	171	232
Number of genes^a^	109	418	33	256	80	89
DAVID	Y.0-X.0		Y.4-X.4		Y.16-X.16	
	Down	Up	Down	Up	Down	Up
DAVID identifiers^b^	70	334	21	194	64	53
Number of clusters	18	104	5	69	19	11
ES > 1.3	3	6	0	7	3	0

^a^adjusted p-value < 0.05 and absolute fold change > 1.3

^b^from the human (hsa) database

**Table 4:** Generic biological terms^a^ from Functional Annotation Clustering (ES > 1.3) between line X and Y

Day 0 - low in Y vs. high in X	
Generic Term^a^	ES
serine proteinase/complement/resp. wounding	1.57
Protease/peptidase activity	1.47
LIM/Zinc finger	1.47
Day 0 - high in Y vs. low in X	
Generic Term	ES
mitochondrial	2.87
organelle lumen (nitracellular/nucleus)	2.84
intracellular protein transport	2.46
protein localization	2.3
mitochondrion outer membrane	1.52
nuclear pore	1.35
Day 4 - high in Y vs. low in X	
Generic Term	ES
translation	2.28
lumen/nucleus	2.15
mitochondrion	1.79
mitochondrion outer membrane	1.72
lipase activity	1.32
regulation of translation	1.3
cytoskeleton/microtubule	1.3
Day 16 - low in Y vs. high in X	
Generic Term	ES
ion transport	2.46
channel activity	1.58
anion transport	1.34

^a^Manually curated the database hits per cluster into as few as possible representative key words

**Fig. 5:**
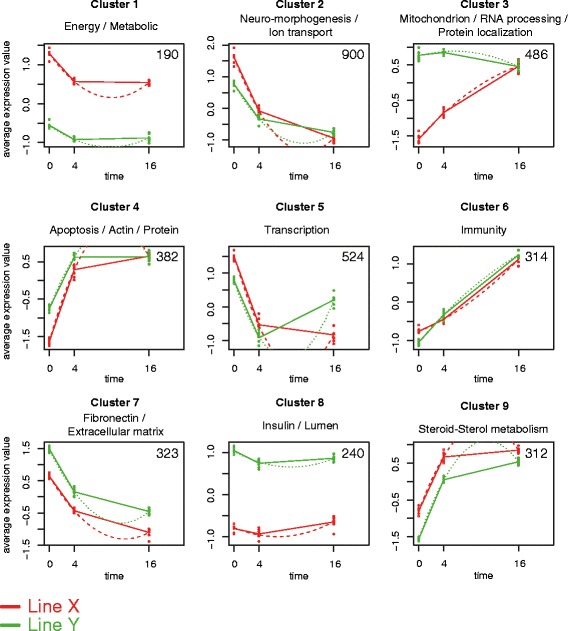
Differences in temporal expression patterns between two genetically different chicken lines by regression based analysis. Nine clusters were obtained after using regression based analysis (maSigPro). Each cluster consists of multiple probes/genes (number of probes/genes shown in top-right corner) displaying differential temporal gene expression patterns between line X (*red line*) and Y (*green line*). The 8 dots per time point and per chicken line represent the average expression value of a pool of 10 chicken. In each graph, he x-axis represents time in days and the y-axis represents the average expression value. Solid lines are the average expression values of the eight pools and dotted/striped lines are predictions from the regression based analysis (maSigPro). The genes in each cluster were used as input for functional analysis (DAVID), and representative key words are shown for each cluster above the graph

To investigate the differences in temporal gene expression patterns between the lines, a regression based approach was performed (R-package maSigPro). This resulted in 3671 probes, corresponding to 1922 genes, that had different temporal expression patterns over time. The next step was to identify (dis)similar temporal expression patterns between the lines, therefore ‘soft’ clustering of the genes was performed (R-package MFuzz). Nine clusters were generated, each displaying a particular expression pattern in time for one or both lines (Table 5). Visualization of these cluster is shown in Fig. 5, were red lines depict line X and green lines depict line Y. Furthermore, to investigate the function of the collection of genes in a certain cluster, functional annotation clustering was performed and the results are depicted in Fig. 5.

**Table 5:** Results of soft clustering of genes displaying a different temporal expression pattern when comparing line X and Y

Cluster	1	2	3	4	5	6	7	8	9	Total
Number of probes	190	900	486	382	524	314	323	240	312	3,671
Number of genes	99	371	295	274	199	156	189	123	216	1,922

## Discussion

In this study two genetically different broiler lines were compared, which were known to differ in their immunological responses towards bacterial infections and the pathology and severity of the induced diseases [29–35]. However, these broiler lines did not differ in any growth characteristics. Thus these broiler lines may differ in the basic level of immune competence. Currently it is not known on what level the two lines deviate from each other and what the underlying mechanisms are for their difference in immunological properties. It is known that programming of the innate as well as the adaptive immune system occurs mainly at young age and the microbial species in the gastro-intestinal tract play a major role in shaping the immune system and the development of the intestinal barrier functions [36, 37]. Compared to human and mice, in chicken not much is known about the impact of genotypic variation on early life microbial colonization in relation to the functional development of the gut. The objective of this study was to investigate the impact of the genetic variation between the two broiler lines on both microbial colonization of the gut and functional development of intestinal tissue. The microbial communities of the two lines (X and Y) differ in composition, but have similar levels of diversity. Also the intestinal gene expression patterns showed marked differences between the two broiler lines, especially at days 0 and 4.

### Microbiota differences in two genetically different broiler lines

In this experiment both broiler lines were hatched at the same hatchery and before starting this experiment the chambers were disinfected with the same protocols. Furthermore, both broiler lines were hatched and reared at the same time (in separate pens) and chickens were exposed to the same environment and nutrition This might explain the small differences observed in the diversity of microbiota, this in agreement with the concept that environmental microbiota are the first colonizers [20, 38]. The composition of microbiota is dependent on the circumstances in the gut and therefore it seems logical to assume that host genetic factors have a bigger impact on microbiota composition than on microbiota diversity. Therefore we hypothesize that the observed difference in microbiota composition over time is due to the fact that these broilers are genetically different. The importance of host genetic factors has already been observed in mice and humans [22, 39, 40]. Since the differences between the lines X and Y in microbiota composition are already demonstrable at the early life stage of the birds, this may have impact on immune programming and the activity of the immune system at later life stages. This hypothesis is in agreement with previous observation with these lines that their immunological responses upon challenge are quite different [29–35]. These studies showed that line Y was more immunological responsive and resistant against both Gram-negative (*Salmonella*) and Gram-positive bacteria (vancomycin-resistant *Enterococcus gallinarum*), as well as parasitic protozoan (*Eimeria tenella*). In recent work, they have proven that chickens with a higher phenotype of key pro-inflammatory mediators (IL-6, CXCLi2 (IL8L2), and MIP family CC chemokine CCLi2) were naturally more resistant against *Salmonella* [41]. The latter shows that it is possible to select for natural resistance against pathogens, here we want to understand the dynamics and kinetics in early development in more detail in order to improve the resistance against pathogens in chicken.

In order to understand the dynamics and kinetics of the colonization, we zoomed in on the major differences in microbiota composition between the lines included *Enterococci*, *Lactobacilli*, and *Escherichia* species. *Enterococci* belong to the lactic acid bacteria, members of the gastro-intestinal tract of hosts, due to the fact that they can colonize a range of hosts including *Caenorhabditis elegans*, insects, reptiles, birds, and mammals [42, 43]. *Enterococci faecalis and E. faecium* are the two most abundant commensals in humans. However, these species are also potential pathogens and contain intrinsic (and acquired) mechanisms for antibiotic resistance and virulence factors (reviewed in [44]) and for this reason they are now labelled as important clinical pathogens. Their specific contribution to the programming and shaping of the immune system is thus far not known.

*Escherichia*, facultative anaerobe bacteria, are another genus that is dominant in early life of these broilers. In adult pigs they have shown that high numbers *Escherichia*, mostly *Escherichia coli*, appear in the gastro-intestinal tract, nonetheless the health status was not affected in these pigs [45]. Although the role of commensal *E. coli* in the gastrointestinal tract is not yet fully understood, we need to acknowledge that *E. coli* is dominant in early life and could fulfil an important role in the development of the gut ecosystem.

*Lactobacilli* are characterized as beneficial for health, mainly because they produce lactic acid which inhibits the growth of (putative) pathogens. Different Lactobacillus strains are already identified that exclude other (pathogenic) bacteria by competitive exclusion or enhancing immunity. For example, *Lactobacillus salivarius* prevents *Salmonella enteritidis* colonization CTC2197 [46]. It has also been shown that a cocktail of bacteria, including *Lactobacillus acidophilus*, *Lactobacillus casei*, *Bifido bacterium bifidium*, and *Enterococcus faecium*, enhances mucosal immunity against *Eimeria acervulina* [47]. Another example is the *Lactobacillus johnsonii* FI9785 bacteria which excludes bacterial pathogens [48]. However, *Lactobacilli* can become pathobionts, but this is mostly in immuno-compromised or elderly people [49]. Different strains of *Lactobacilli* are also used as probiotics to improve the health status of humans [50–53] and chickens [54–56]. This shows, that a higher abundance of (particular) *Lactobacilli* strains in the gut may affect health status beneficially [54–56], either by reducing the pathogen load and/or strengthening the immune defence against infections. In this research, the time factor is predominantly present throughout the data, i.e. the colonization of the *Lactobacilli* from 0-0.1 % ARC at day 0 to 84-88 % ARC at day 4. This high relative contribution of *Lactobacilli* over time may also be important for the immune development in the gut. Only small differences between line X and Y are observed, where line X has slightly higher abundance at day 0, which could be associated to the high activity of immune genes.

### Different temporal gene expression patterns between broiler lines

The expression of many genes and processes in the intestinal tissue differed between the two broiler lines. The most dominant processes were related to cell cycle and proliferation. Another cluster was associated to immunity, whereas other clusters could not be linked to immunity or barrier function, such as metabolic and transcriptional processes.

The link of genes being involved in cell cycle and proliferation is shown in several clusters (3, 4, and 7). More specifically these clusters were associated to apoptosis, extracellular matrix, and mitochondrion. These results show that cell proliferation/apoptosis is a main difference between these lines, especially in early age, these processes may be directly linked to the barrier function of the gut. It has been estimated that every 3-5 days the gut is renewed [57], tight regulation of cell death (apoptosis) and proliferation of epithelial cells is paramount to ensure structural integrity in the gut [58]. In chicken multiple interventions, including fasting, diet, thermal conditioning, and stress, already have been described that affect epithelial structure, growth, and function [59–62].

Moreover, the intestinal barrier has an important role in maintaining homeostasis and when dysfunction of the intestinal barrier occurs this is associated to several gastrointestinal diseases, including inflammatory bowel disease, food intolerance, and celiac disease [63–66]. Compared to line X, line Y showed higher average expression of cell cycle and proliferation related processes which may contribute to the immune phenotype observed, higher resistance against bacterial infections. This data suggests that a heightened status of the barrier function in early life could lead to less mortality. This could also be reflected in the lower activity of immune genes at day 0 in line Y, indicating that the immune programming might be delayed.

We were surprised to see limited effects on immune related processes. Only one cluster differing between line X and Y was associated with immunity, one could expect more because these lines differ in resistance against bacterial infection and showed deviating gene expression after an immune challenge [29–35]. This immunity cluster only differed at day 0, where line X showed higher expression compared to line Y and from day 4 to 16 a similar expression pattern was observed. This immunological expression pattern in time was already observed in earlier work of our group and others [16–18]. This suggests that overall immune development in these broiler lines were comparable except at early stage. Thus, in order for chickens to survive the turbulent start of life which is accompanied with the microbial colonization and gut differentiation, chickens may either invest in the immune programming or the barrier function at an early stage.

## Conclusions

- Both colonization of gut microbiota and functional processes are influenced by the genetic background
- Microbiota composition diverges over time
- Intestinal functional processes and corresponding gene expression are mainly affected in early life between two genetically different chicken lines
- Temporal differences between these two lines suggest different coping mechanisms in early life

## Methods

### Experimental design

1-day-old chicks (Lines X and Y) were housed in a floor pen system in which the chicks had *ad libitum* access to feed and water. At days 0, 4 and 16, 80 birds of each line X and line Y were sacrificed for tissue sampling, in total 240 birds per line. Subsequently, samples from these 80 birds were pooled in 8 pools of ten birds. Samples were pooled because our main interest was in the generic processes at the population level of these genetically divergent broiler lines. We acknowledge that we lose the ability to interpret the data on an individual level. This pooling strategy, 8 pools of 10 chicken, enabled us to identify smaller differences between the two genetically different broiler lines compared to analysing 8 individual chickens. The absolute difference in the average of individual samples appointed significant was 1.3, whereas in the pooling strategy this was reduced to 0.4.

The Feed Conversion Ratio (FCR) was calculated by dividing the feed intake for the time-period of interest by the average daily gain for the time-period of interest. The following time-periods were analysed 0-9, 9-16, 16-23, and 23-36, and each time the FCR for line X was subtracted from line Y to investigate the difference between the broiler lines.

### Ethics statement

This animal experiment was approved by the institutional animal experiment committee “Dier Experimenten Commissie (DEC) Lelystad” (accession number 2013035.b), in accordance with the Dutch regulations on animal experiments.

### Microbiota analysis

#### DNA extraction

Jejunal content was snap frozen in liquid nitrogen and stored at -80 °C. For the microbial DNA extraction the following protocol was used. Jejunal content was mixed 1:1 with PBS and vortexed, spun for 5 min (300 g) at 4 °C. The supernatant was transferred to a new tube and spun for 10 min (9000 g) at 4 °C, thereafter supernatant was removed. Subsequently the QIAamp DNA Stool Mini Kit protocol was used as described by the manufacturer (https://www.qiagen.com/nl/products/catalog/sample-technologies/dnasample-technologies/genomic-dna/qiaamp-dna-stool-mini-kit/). The samples were eluted in 100 μl of the (provided) elute buffer and thereafter an optical density measurement was performed to check the DNA quality on Nanodrop (Agilent Technologies).

#### V3 PCR

Oligo V3_F, with sequence; CCTACGGGAGGCAGCAG and oligo V3_R with sequence; ATTACCGCGGCTGCTGG.

The following PCR program was ran: Step 1 98 °C for 2 min. Step 2 98 °C for 10s, step 3 55 °C for 30s, and step 4 72 °C for 10s, step 5 72 °C for 7 min. Where step 2-4 were repeated for 15 times. All PCR products were subjected to quality control by running 5 μl of PCR product on a 2 % E-Gel® Agarose Gel Electrophoresis System (Life Technologies).

#### QIIME

Samples were sequenced by targeted-amplicon 16S sequencing on the MiSeq (Additional file 1: Table S1 shows the reads per sample and Additional file 1: Table S2 shows the summary per broiler line at each time-point) and analysed for taxonomy profile per sample with clustering by profile by using QIIME [67]. Standard assembly based on amplicon, with primer removal was performed. For Quality filtration the following settings were used: 1) > Q20 and 2) amplicons >100 bases. For the data analysis pseudoreads were clustered into operational taxonomic units (OTUs) per sample at 97 % similarity and OTU-representative sequences were aligned against the aligned Greengenes core set (13_8 release) [68, 69]. Singletons were removed, as well as chimeras, the latter with Chimeraslayer [70].

#### Statistical analysis

The biodiversity was calculated by the vegan package (http://cran.r-project.org/web/packages/vegan/) within the R environment, by employing the Shannon diversity index. The richness and evenness data (together with the diversity) are depicted in Additional file 1: Tables S3, S4 and S5. The Redundancy analysis (RDA) was also performed by using the vegan package, the following model was ran on the family level microbiota data: y = Time + Treatment + Time* Treatment + error. Furthermore, statistical significance testing for over- and under-representation of the bacterial groups was made at the family level by performing the Wilcoxon signed-rank test, and p-values were converted to false discovery rate (FDR) values to correct for multiple testing.

### Transcriptomic analysis

#### RNA extraction tissue

Total RNA was extracted from 50 to 100 mg jejunum tissue. All samples were homogenised using the TisuPrep Homogenizer Omni TP TH220P) in TRizol reagent (Life Technologies) as recommended by the manufacturer with minor modifications. The homogenised tissue samples were dissolved in 5 ml of TRizol reagent. After centrifugation the supernatant was transferred to a fresh tube. Subsequently a phase separation with chloroform was performed as described by the manufacturer Life Technologies. The RNA was precipitated and dissolved and quantified by absorbance measurements at 260 nm. Quality Control was performed using the Agilent Bioanalyser.

#### Labelling, hybridization, scanning and feature extraction

Labelling of RNA was done as recommended by Agilent Technologies using the One-Color Microarray-Based Gene Expression Analysis Low input Quick Amp Labelling. The input was 10 ng of total RNA and 600 ng of labelled cRNA was used for hybridization on the eight pack array (Agilent 049577 chicken array). Hybridization was performed as described in the One-Color Microarray-Based Gene Expression Analysis Low input Quick Amp Labelling protocol from Agilent in the hybridization oven (G2545A hybridization Oven Agilent Technologies). The hybridization temperature was 65 °C with rotation speed 10 rpm for 17 h. After 17 h the arrays were washed as described in the One-Color Microarray-Based Gene Expression Analysis Low input Quick Amp Labelling protocol from Agilent. The arrays were scanned using the DNA microarray scanner with Surescan high resolution Technology from Agilent Technologies. Agilent Scan Control with resolution of 5 μm, 16 bits and PMT of 100 %. Feature extraction was performed using protocol 10.7.3.1 (v10.7) for 1 colour gene expression.

#### Data analysis

The gene expression data (GEO accession number GSE65042) were analysed by using R (v3.0.2) by executing different packages, including LIMMA [71] and arrayQualityMetrics [72]. The data were read in and background corrected (method = “normexp” and offset = 1) with functions from the R package LIMMA [71] from Bioconductor [73]. Quantile normalisation of the data was done between arrays. The duplicate probes mapping to the same gene were averaged (‘avereps’) and subsequently the lower percentile of probes were removed in a three-step procedure, 1) get the highest of the dark spots to get a base value, 2) multiply by 1.1, and 3) the gene/probe must be expressed in each of the samples in the experimental condition [74].

#### Statistical and functional genomics analysis

To test the differences between the chicken lines (X and Y) on each day separately, the following contrasts, Y.0-X0, Y.4-X.4, and Y.16-X.16, were generated within the LIMMA package [71]. DAVID [75] was used to perform Functional Annotation Clustering (FAC) for the three different contrasts, i.e. Y.0-X0, Y.4-X.4, and Y.16-X.16. The up- and down-regulated genes were separately analysed.

To investigate the temporal patterns for each chicken line and to find similarities and dissimilarities, we have performed a maSigPro analysis with subsequent soft clustering (MFuzz package). Clustering of data is an important bioinformatic tool in transcriptomic time-analysis and can uncover structures buried in these large transcriptomic datasets. Soft clustering is less sensitive to noise, compared to hard clustering and soft clustering has two main advantages, by generating how well clusters represent genes and the overall relation between clusters [76, 77]. MaSigPro is a R-package [78], especially generated to handle short time-series microarray gene expression data and to find genes, based on a regression based approach, which have significantly different expression profiles between treatments. The following settings were used, in p.vector the Q value was set at 0.01 and multiple testing correction was performed by using Benjamini Hochberg with minimum observation of 20. For T.fit, which makes a stepwise regression fit for time series gene expression experiments, the ‘backward’ step method was used with ‘alfa’ at 0.05. Filtering of genes based on r-square (>0.8) was performed and ‘vars’ was set at ‘each’, generating as many significant genes extractions as variables in the general regression model.

### Availability of supporting data

The transcriptomics data discussed in this study have been deposited in NCBI Gene Expression Omnibus (GEO; http://www.ncbi.nlm.nih.gov/geo/), and are accessible through GEO series accession no. GSE65042 (http://www.ncbi.nlm.nih.gov/geo/query/acc.cgi?acc=GSE65042).

The microbiota data discusses in this study have been deposited in figshare (http://figshare.com/), and are accessible through http://dx.doi.org/10.6084/m9.figshare.1406903.

## Additional file

Additional file 1:
**Suplementary Tables.** **Table S1.** MiSeq reads per sample. **Table S2.** MiSeq reads summary. **Table S3.** Species diversity measurements. **Table S4.** Species diversity summary. **Table S5.** Statistical testing for differences in species diversity measurements between broiler lines at each time-point.
