# Supplementary material for: Early life microbial colonization of the gut and intestinal development differ between genetically divergent broiler lines
Source: BMC Genomics. 2015 May 28;16(1):418. doi: 10.1186/s12864-015-1646-6 (PMC4446945; doi:10.1186/s12864-015-1646-6)
Supplement: Additional file 1: — Suplementary Tables. Table S1. MiSeq reads per sample. Table S2. MiSeq reads summary. Table S3. Species diversity measurements. Table S4. Species diversity summary. Table S5. Statistical testing for differences in species diversity measurements between broiler lines at each time-point. [file 12864_2015_1646_MOESM1_ESM.doc]

Supplementary Tables

Table S1 MiSeq reads per sample

| **Pool** | **Line** | **Age** | **Line.Age** | **Reads** | **Pool** | **Line** | **Age** | **Line.Age** | **Reads** |
| --- | --- | --- | --- | --- | --- | --- | --- | --- | --- |
| X2 | X | 0 | X0 | 1021220 | Y2 | Y | 0 | Y0 | 1516049 |
| X3 | X | 0 | X0 | 883461 | Y3 | Y | 0 | Y0 | 1646448 |
| X4 | X | 0 | X0 | 1217463 | Y4 | Y | 0 | Y0 | 1113153 |
| X5 | X | 0 | X0 | 858707 | Y5 | Y | 0 | Y0 | 1446494 |
| X6 | X | 0 | X0 | 1048364 | Y6 | Y | 0 | Y0 | 1746496 |
| X7 | X | 0 | X0 | 1032836 | Y7 | Y | 0 | Y0 | 924875 |
| X8 | X | 0 | X0 | 817078 | Y8 | Y | 0 | Y0 | 1038047 |
| X9 | X | 4 | X4 | 959948 | Y9 | Y | 4 | Y4 | 1211536 |
| X10 | X | 4 | X4 | 1668735 | Y10 | Y | 4 | Y4 | 1291354 |
| X11 | X | 4 | X4 | 1589937 | Y11 | Y | 4 | Y4 | 1213121 |
| X12 | X | 4 | X4 | 758785 | Y12 | Y | 4 | Y4 | 1350627 |
| X13 | X | 4 | X4 | 1670203 | Y13 | Y | 4 | Y4 | 1232810 |
| X14 | X | 4 | X4 | 1565244 | Y15 | Y | 4 | Y4 | 1449059 |
| X15 | X | 4 | X4 | 873221 | Y16 | Y | 4 | Y4 | 1471600 |
| X16 | X | 4 | X4 | 889724 | Y17 | Y | 16 | Y16 | 911681 |
| X17 | X | 16 | X16 | 1298821 | Y18 | Y | 16 | Y16 | 1368195 |
| X18 | X | 16 | X16 | 1249677 | Y19 | Y | 16 | Y16 | 769562 |
| X19 | X | 16 | X16 | 1357991 | Y20 | Y | 16 | Y16 | 716567 |
| X20 | X | 16 | X16 | 704148 | Y21 | Y | 16 | Y16 | 1506785 |
| X21 | X | 16 | X16 | 826374 | Y22 | Y | 16 | Y16 | 1416140 |
| X22 | X | 16 | X16 | 1348373 | Y23 | Y | 16 | Y16 | 1295903 |
| X23 | X | 16 | X16 | 499229 | Y24 | Y | 16 | Y16 | 1440025 |
| X24 | X | 16 | X16 | 500522 |  | | | | |

Table S2 MiSeq reads summary

| **Line.Age average** | **Reads** | **SEM^1^** |
| --- | --- | --- |
| X0 | 982733 | 52571 |
| X4 | 12469775 | 144170 |
| X16 | 973142 | 134517 |
| Y0 | 1347366 | 121113 |
| Y4 | 1317158 | 41441 |
| Y16 | 1178107 | 114503 |

^1^SEM. standard error of the mean

Table S3 Species diversity measurements

| **Pool** | **Line** | **Age** | **Evenness** | **Richness** | **Diversity^1^** | **Pool** | **Line** | **Age** | **Evenness** | **Richness** | **Diversity** |
| --- | --- | --- | --- | --- | --- | --- | --- | --- | --- | --- | --- |
| X2 | X | 0 | 0.058 | 29 | 0.197 | Y2 | Y | 0 | 0.139 | 76 | 0.604 |
| X3 | X | 0 | 0.060 | 73 | 0.260 | Y3 | Y | 0 | 0.091 | 34 | 0.320 |
| X4 | X | 0 | 0.047 | 47 | 0.184 | Y4 | Y | 0 | 0.247 | 37 | 0.894 |
| X5 | X | 0 | 0.175 | 61 | 0.719 | Y5 | Y | 0 | 0.190 | 87 | 0.848 |
| X6 | X | 0 | 0.212 | 56 | 0.853 | Y6 | Y | 0 | 0.106 | 105 | 0.494 |
| X7 | X | 0 | 0.106 | 42 | 0.397 | Y7 | Y | 0 | 0.148 | 90 | 0.666 |
| X8 | X | 0 | 0.155 | 82 | 0.682 | Y8 | Y | 0 | 0.194 | 34 | 0.685 |
| X9 | X | 4 | 0.109 | 121 | 0.523 | Y9 | Y | 4 | 0.125 | 57 | 0.508 |
| X10 | X | 4 | 0.108 | 78 | 0.473 | Y10 | Y | 4 | 0.280 | 68 | 1.184 |
| X11 | X | 4 | 0.121 | 73 | 0.520 | Y11 | Y | 4 | 0.192 | 69 | 0.815 |
| X12 | X | 4 | 0.206 | 90 | 0.930 | Y12 | Y | 4 | 0.115 | 62 | 0.477 |
| X13 | X | 4 | 0.086 | 98 | 0.395 | Y13 | Y | 4 | 0.238 | 82 | 1.051 |
| X14 | X | 4 | 0.238 | 80 | 1.044 | Y15 | Y | 4 | 0.185 | 75 | 0.798 |
| X15 | X | 4 | 0.188 | 118 | 0.899 | Y16 | Y | 4 | 0.137 | 73 | 0.590 |
| X16 | X | 4 | 0.221 | 101 | 1.020 | Y17 | Y | 16 | 0.321 | 196 | 1.698 |
| X17 | X | 16 | 0.123 | 90 | 0.556 | Y18 | Y | 16 | 0.206 | 122 | 0.992 |
| X18 | X | 16 | 0.161 | 78 | 0.702 | Y19 | Y | 16 | 0.238 | 142 | 1.181 |
| X19 | X | 16 | 0.126 | 95 | 0.577 | Y20 | Y | 16 | 0.207 | 152 | 1.042 |
| X20 | X | 16 | 0.393 | 109 | 1.847 | Y21 | Y | 16 | 0.229 | 125 | 1.107 |
| X21 | X | 16 | 0.404 | 112 | 1.909 | Y22 | Y | 16 | 0.187 | 120 | 0.895 |
| X22 | X | 16 | 0.223 | 116 | 1.060 | Y23 | Y | 16 | 0.281 | 156 | 1.419 |
| X23 | X | 16 | 0.424 | 108 | 1.985 | Y24 | Y | 16 | 0.251 | 126 | 1.217 |
| X24 | X | 16 | 0.438 | 104 | 2.036 | Y24 |  |  |  |  |  |

^1^Diversity calculated by Shannon-index

Table S4 Species diversity summary

| **Line.Age** | **Evenness** | **SEM^1^** | **Richness** | **SEM** | **Diversity^2^** | **SEM** |
| --- | --- | --- | --- | --- | --- | --- |
| X0 | 0.12 | 0.02 | 55.71 | 6.89 | 0.47 | 0.10 |
| X4 | 0.16 | 0.02 | 94.88 | 6.37 | 0.73 | 0.10 |
| X16 | 0.29 | 0.05 | 101.50 | 4.54 | 1.33 | 0.24 |
| Y0 | 0.16 | 0.02 | 66.14 | 11.47 | 0.64 | 0.07 |
| Y4 | 0.18 | 0.02 | 69.43 | 3.14 | 0.77 | 0.10 |
| Y16 | 0.24 | 0.02 | 142.38 | 9.10 | 1.19 | 0.09 |

^1^SEM, standard error of the mean; ^2^Diversity calculated by Shannon-index

Table S5 Statistical testing for differences in species diversity measurements between broiler lines at each time-point

| **Evenness** | | **Richness** | | **Diversity** | |
| --- | --- | --- | --- | --- | --- |
| **T-test^1^** | **p-value** | **T-test** | **p-value** | **T-test** | **p-value** |
| Y0 vs. X0 | 0.206 | Y0 vs. X0 | 0.451 | Y0 vs. X0 | 0.201 |
| Y4 vs. X4 | 0.490 | Y4 vs. X4 | 0.005 | Y4 vs. X4 | 0.732 |
| Y16 vs. X16 | 0.387 | Y16 vs. X16 | 0.001 | Y16 vs. X16 | 0.590 |

^1^2-sided homoscedastic Student’s t-test
